# The Hidden Threat: Endocrine Disruptors and Their Impact on Insulin Resistance

**DOI:** 10.7759/cureus.47282

**Published:** 2023-10-18

**Authors:** Mehak Dagar, Priya Kumari, Agha Muhammad Wali Mirza, Shivani Singh, Noor U Ain, Zainab Munir, Tamleel Javed, Muhammad Furqan Ismat Virk, Saleha Javed, Farwa Haider Qizilbash, Anil KC, Chukwuyem Ekhator, Sophia B Bellegarde

**Affiliations:** 1 Internal Medicine, Himalayan Institute of Medical Sciences, New Delhi, IND; 2 Medicine, Jinnah Postgraduate Medical Centre, Karachi, PAK; 3 Internal Medicine, Jinnah Medical and Dental College, Karachi, PAK; 4 Medicine, MediCiti Institute of Medical Sciences, Hyderabad, IND; 5 Medicine, Mayo Hospital, Lahore, PAK; 6 Medicine, King Edward Medical University, Lahore, PAK; 7 Emergency Department, Imran Idrees Teaching Hospital, Sialkot, PAK; 8 Paediatrics, ABWA Hospital and Research Centre, Faisalabad, PAK; 9 Emergency Department, Sheikh Zayed Hospital, Rahim Yar Khan, PAK; 10 Medicine, Dow Medical College and Civil Hospital, Karachi, PAK; 11 Medicine and Surgery, Patan Academy of Health Sciences, Kathmandu, NPL; 12 Medicine, California Institute of Behavioral Neurosciences and Psychology, Fairfield, USA; 13 Neuro-Oncology, New York Institute of Technology College of Osteopathic Medicine, Old Westbury, USA; 14 Pathology and Laboratory Medicine, American University of Antigua, Coolidge, ATG

**Keywords:** heavy metals, pesticides, polycyclic aromatic hydrocarbons, environmental endocrine-disrupting chemicals, triclosan, bisphenol a, perfluoroalkyl substances, phthalates, insulin resistance, endocrine disruptors

## Abstract

The association between Insulin resistance, a global health issue, and endocrine disruptors (EDCs), chemicals interfering with the endocrine system, has sparked concern in the scientific community. This article provides a comprehensive review of the existing literature regarding the intricate relationship between EDCs and insulin resistance. Phthalates, commonly found in consumer products, are well-established EDCs with documented effects on insulin-signaling pathways and metabolic processes. Epidemiological studies have connected phthalate exposure to an increased risk of type 2 diabetes mellitus (T2DM). Perfluoroalkyl substances (PFAS), persistent synthetic compounds, have shown inconsistent associations with T2DM in epidemiological research. However, studies suggest that PFAS may influence insulin resistance and overall metabolic health, with varying effects depending on specific PFAS molecules and study populations. Bisphenol A (BPA), found in plastics and resins, has emerged as a concern for glucose regulation and insulin resistance. Research has linked BPA exposure to T2DM, altered insulin release, obesity, and changes in the mass and function of insulin-secreting β-cells. Triclosan, an antibacterial agent in personal care products, exhibits gender-specific associations with T2DM risk. It may impact gut microbiota, thyroid hormones, obesity, and inflammation, raising concerns about its effects on metabolic health. Furthermore, environmental EDCs like polycyclic aromatic hydrocarbons, pesticides, and heavy metals have demonstrated associations with T2DM, insulin resistance, hypertension, and obesity. Occupational exposure to specific pesticides and heavy metals has been linked to metabolic abnormalities.

## Introduction and background

Insulin resistance (IR), a metabolic disorder characterized by the reduced ability of the body to respond to insulin, has become a significant health concern worldwide [1]. On the other hand, endocrine disruptors (EDCs), a group of chemicals that interfere with the endocrine system, have gained attention due to their potential adverse effects on health [2]. The intersection of these two areas of study presents a complex and compelling subject of investigation.

Insulin, a hormone secreted by the pancreas, plays a vital role in regulating blood glucose levels by facilitating the uptake of glucose into cells. IR occurs when cells become less responsive to insulin, leading to elevated blood sugar levels. This condition is often associated with obesity, type 2 diabetes mellitus (T2DM), and various cardiovascular complications [3]. Understanding the underlying mechanisms of IR is crucial for addressing these widespread health issues. EDCs are a diverse group of synthetic and natural chemicals that can interfere with the endocrine system's normal function. They are found in everyday products such as plastics, pesticides, and personal care items. These disruptors can mimic or block hormones in the body, leading to hormonal imbalances. The consequences of exposure to EDCs have been linked to a wide range of health problems, including reproductive disorders, developmental issues, and certain cancers [4].

The significance of unraveling the molecular connections between IR and EDCs lies in the potential to shed light on complex health challenges. By understanding how these disruptors affect insulin signaling pathways and metabolic processes, we can develop strategies to mitigate their impact and improve public health. While studies have independently investigated IR and EDCs, there is a noticeable research gap in comprehensively exploring how these two areas intersect at the molecular level. Understanding the intricate web of molecular interactions between EDCs and IR is essential for developing effective prevention and intervention strategies.

## Review

IR and endocrine disruption

EDCs have garnered increasing attention for their potential role in disrupting the endocrine system, which regulates hormones and plays a vital role in various physiological processes. While much of the research on EDCs has focused on their impact on reproductive health and development, emerging evidence suggests that these chemicals may also influence metabolic health, particularly in children [5]. This article reviews the available literature linking background exposure to EDCs with IR in both adults and children. The literature addressing the link between various EDCs and IR was searched using PubMed and Google Scholar. The results are summarized and discussed in this review.

IR is a key player in the development of metabolic disorders like obesity and T2DM. It is characterized by a decreased tissue response to insulin, leading to impaired glucose utilization, increased hepatic glucose output, and altered protein and lipid metabolism. While genetic factors and lifestyle choices contribute to IR, emerging research suggests that EDCs may also play a role in its development. Numerous epidemiological studies have investigated the association between EDC exposure and IR [6-8]. The reviewed studies have identified several classes of EDCs that may contribute to this metabolic condition, including pesticides, polychlorinated biphenyls, bisphenol A (BPA), phthalates, polycyclic aromatic hydrocarbons (PAH), and dioxins. Specifically, cross-sectional studies have demonstrated statistical links between exposure to these EDCs and IR in human populations [3,5,6,9]. The prevalence of diabetes and obesity appears to increase in individuals with higher levels of EDC exposure. These findings are concerning and suggest that environmental factors, such as EDCs, may be contributing to the rising rates of metabolic disorders.

Phthalates

Phthalates are a group of chemicals known as esters of 1,2-dibenzene dicarboxylic acid [10]. They are flexible molecules utilized in a wide range of consumer goods, including cosmetics, medicines, and polymers, thanks to their generic chemical structure. Phthalates are classified as EDCs because they can disrupt the body's hormonal systems [11]. Phthalates can enter the body by ingestion, inhalation, or skin absorption, making exposure to these substances among pregnant women, infants, and children across the world practically ubiquitous [12-15]. The ability of phthalates to pass through the placenta and expose the fetus to them is very concerning [16]. Phthalates are quickly converted into their corresponding mono-ester forms once within the body. Low-molecular-weight phthalates, including di-ethyl phthalate (DEP), di-n-butyl phthalate, and di-iso-butyl phthalate, are mostly excreted in urine as glucuronide or sulfate-conjugated hydrolytic monoesters. On the contrary, the hydrolytic metabolites of di-2-ethylhexyl phthalate (DEHP) and mono-2-ethylhexyl phthalate (MEHP) go through further enzymatic oxidation before being conjugated and excreted. Though phthalates have biological half-lives that are shorter than 24 hours, they are frequently exposed again and again [17,18]. As a result, urine biospecimens are frequently utilized to properly determine phthalate exposure.

Phthalates have the capacity to affect how hormones such as androgens, thyroid hormones, and glucocorticoids function and are metabolized inside the body. Additionally, they have the ability to bind to several nuclear receptors, which renders them slightly estrogenic and affects reproductive functions [19]. In addition, high-molecular-weight phthalates, such as DEHP, interfere with the function of peroxisome proliferator-activated receptors, which are crucial for lipid metabolism and energy balance, and thyroid receptors, which alter metabolism [20]. Furthermore, phthalates affect liver X receptors, major regulators of glucose, fatty acid, and cholesterol homeostasis, as well as the aryl hydrocarbon receptor, which is essential for the metabolism of xenobiotics [21]. The potential for phthalates to interfere with metabolic processes in the body is highlighted by their wide range of effects on hormone systems and receptors.

The relationship between phthalate exposure and IR in humans has been investigated in a number of studies. Particularly in middle-aged women, urinary phthalate levels have been associated with an elevated risk of T2DM [22]. In several groups, including the general adult population in Korea, adult males in Shanghai, and middle-aged women in studies like the Nurses' Health Study (NHS) and the Study of Women's Health Across the Nation (SWAN) study, phthalate metabolites have been positively related to the risk of T2DM [22-25]. Urinary DEHP levels have been linked to metabolic syndrome elements in teenagers, while other phthalates have been linked to IR [26]. A meta-analysis supports the relationship between phthalates and the risk of T2DM [27]. According to estimates, phthalate exposure may be a factor in the thousands of new instances of diabetes that occur each year in Europe and the US [28].

Studies on animals shed light on the possible processes that could underlie the link between phthalates and IR. According to studies done on rodents, exposure to DEHP can lower the amount of pancreatic insulin produced and affect the shape of pancreatic β-cells, which lowers the number of these cells and alters blood sugar levels [29]. Furthermore, mice exposed to phthalates have been shown to have higher body weights, fatty livers, and altered glucose tolerance [30,31].

Reduced expression of genes associated with insulin and increased oxidative stress have been shown in conjunction with inhibitory effects on β-cell activity, which may help explain decreased insulin production. Furthermore, exposure to phthalates has been linked to decreased β-cell survival through apoptosis, cytotoxicity, altered signaling cascades, and endoplasmic reticulum stress, among other mechanisms [32-34].

Per- and polyfluoroalkyl substances 

A class of synthetic compounds called per- and polyfluoroalkyl substances (PFAS) are renowned for their extraordinary resistance to biological, chemical, and thermal degradation [35,36]. Many consumer items, including nonstick cookware, stain-resistant textile coatings, food container linings, fire-fighting foam, floor polishes, and industrial surfactants, utilize these synthetic substances [36,37]. PFAS are characterized by the presence of multiple fluorine atoms attached to an alkyl chain, forming a strong C-F chemical bond. With biological half-lives ranging from 3.8 to 7.3 years, this unique chemical structure leads to their extraordinary persistence in the environment and bioaccumulation over time in human tissues. Concerns have been raised regarding the possible negative consequences of PFAS exposure due to its pervasiveness and long-lasting nature. Research on the harmful health impacts of PFAS exposure has increased over the past two decades, exposing connections to a variety of chronic disorders, including immunotoxicity, cardiometabolic disease, effects on development and reproduction, and even cancer [8,38]. The probable link between PFAS exposure and IR, a key component in the emergence of T2DM, has come to light more recently.

Numerous studies have revealed a favorable relationship between blood PFAS levels and certain T2DM risk markers. For example, in a study by Domazet et al., it was discovered that perfluorooctanoic acid (PFOA) exposure during childhood was linked to diminished β-cell function, a crucial element in the development of T2DM [39]. According to a study by Kim et al., perfluorooctanesulfonic acid (PFOS) and perfluorododecanoic acid (PFDoDA) were favorably correlated with the IR biomarker, Homeostatic Model Assessment for IR (HOMA-IR) [40]. According to Cardenas et al., doubling the concentrations of PFOS and PFOA was associated with increased HOMA-IR, HOMA-β, fasting proinsulin, and glycated hemoglobin (HbA1c) levels [41]. Significantly favorable correlations between PFAS exposure and fasting glucose and HbA1c levels were found in a 2019 study in Tianjin, China [42]. Significant favorable relationships between different PFAS and fasting blood sugar, fasting insulin, and HOMA-IR were discovered by Zeeshan et al. in a study in 2021 [43]. PFOS exposure was linked to reduced insulin sensitivity and increased insulin secretion, according to a cohort study by Valvi et al. [44].

In contrast, several studies have reported non-significant or inverse associations between PFAS exposure and T2DM risk markers; Liu et al. found that branched PFOA and linear PFOS were significantly associated with decreased fasting glucose, with no significant associations with two-hour glucose, insulin levels, or HOMA-IR [45]. Nelson et al. found no evidence of a connection between exposure to PFAS and HOMA-IR [46]. Fisher et al. found no correlations between PFAS levels and fasting insulin, fasting glucose, or HOMA-IR [47]. Fleisch et al. discovered that lower HOMA-IR was related to greater amounts of PFOA, PFOS, and perfluorodecanoate (PFDeA), especially in females [48]. Fassler et al. observed borderline relationships between PFAS exposure and fasting insulin, IR, and insulin sensitivity [49]. These studies often analyzed populations with lower PFAS exposure levels, potentially contributing to the observed non-significant or inverse associations.

Lundin et al. reported an increased risk of diabetes and diabetes-related deaths in moderately exposed workers from a PFAS-producing company but not in low or high exposures [50]. Other studies have investigated the relationship between PFAS exposure and the incidence of T2DM. Sun et al. discovered a significant correlation between increased T2DM risk in female nurses and higher plasma concentrations of PFOS and PFOA [51]. Zeeshan et al. found that blood concentrations of all PFAS were linked to an elevated incidence of T2DM in adults, with branching PFOS having the highest risk [43]. He et al. found that a significant connection between PFOA exposure and the prevalence of diabetes was seen in males, but not females [52]. These findings suggest that PFAS exposure may influence the development of T2DM, although the strength of the association and gender-specific effects vary between studies.

The complex relationship between PFAS exposure and T2DM risk underscores the challenges of drawing definitive conclusions. Numerous variables, including the particular PFAS molecule under research, exposure levels, demographic characteristics, and study methodology, may contribute to the heterogeneity of results. Furthermore, the non-monotonic dose-response correlations seen in certain studies underline the need for additional in-depth investigation to comprehend the complex effects of PFAS on IR and T2DM risk. Further research is necessary to completely understand the processes behind these relationships.

BPA

BPA is a member of the class of bisphenols and diphenylmethane derivatives and is distinguished by possessing two hydroxyphenyl groups. Consumer goods, including toys, infant bottles, medical equipment, and food and beverage containers, are all made using this substance [53]. Although BPA has generally been regarded as safe by regulatory bodies, new data indicates that it could have negative impacts on glucose homeostasis, pancreatic β-cell function, and metabolic health in general. Due to its potential to leak from food and beverage containers, BPA is typically consumed orally [54]. Inhalation and dermal absorption, however, can further increase exposure, particularly for people handling BPA-containing receipts [55]. Pregnant women, newborns, and kids have all been found to be heavily exposed to BPA in studies on exposure levels.

In 2008, Lang et al. published a cross-sectional study that connected increased urine BPA levels with T2DM and other health problems, marking a key turning point in the investigation of BPA's health impacts [56]. Since then, correlations between BPA exposure and T2DM have been found in a number of cross-sectional investigations [57,58]. Even more alarming, a systematic review and meta-analysis found a correlation between the incidence of T2DM and a number of EDCs, including BPA [59]. Notably, several studies have shown conclusive proof that poor glucose homeostasis, including hyperinsulinemia and IR, is linked to increased urine BPA levels [60,61]. These correlations exhibit sex-dependent effects, with males having higher correlations. It's interesting to note that after consuming a single dosage of BPA, changes in insulin release have been seen in people [62,63]. These results imply that BPA may have different effects depending on age and weight on early-phase insulin release and contribute to IR in both non-obese and obese people. In addition, BPA exposure has been linked to childhood and teenage obesity, a significant risk factor for T2DM. In contrast to bisphenol-F (BPF), which did not have the same impact, BPA substitutes like bisphenol-S (BPS) have also shown links to T2DM [63,64].

Studies on animals, mainly rodents, offer important insights into how BPA affects glucose homeostasis. Even low-dose BPA exposure has been demonstrated to cause IR and hyperinsulinemia in adult rats, possibly through the action of estrogen receptors [65]. BPA has also been linked to hypercholesterolemia and glucose intolerance in rats. These results indicate that BPA can interfere with glucose homeostasis by influencing a number of organs involved in energy metabolism, including the liver, skeletal muscle, and adipose tissue [66]. There have also been reports of transgenerational effects when exposure to BPA during fetal development led to altered glucose metabolism in offspring. These side effects include hyperinsulinemia, IR, and glucose intolerance [67]. This is significant because it indicates that BPA exposure during pregnancy may have an effect on the long-term metabolic health of both mother and offspring. These alterations also coexist with variations in β-cell mass and function.

Triclosan (TCS)

A common antibacterial ingredient called TCS is included in a wide range of consumer goods, including soaps, toothpaste, and other personal care products [68]. Despite its widespread usage, recent studies raise questions regarding the possible negative consequences of TCS exposure on health, including its link to IR and the risk of T2DM. In a manner comparable to BPA, oral and dermal exposure are the main ways TCS is exposed. It is important to note that TCS is not a persistent substance because of its brief biological half-life of less than 24 hours [69]. Following exposure, TCS is mostly eliminated in the urine as conjugates of glucuronide or sulfate [70]. Pregnant women and children are almost always exposed to TCS, according to biomonitoring studies, which highlights the need for investigation into its possible health risks [71].

Recent epidemiological studies, including analysis of the National Health and Nutrition Examination Survey (NHANES) 2013-2014 data, have revealed intriguing associations between TCS exposure and the risk of T2DM, with notable gender-specific differences [72]. Although there is conflicting evidence in the literature about the link between TCS exposure and T2DM, these studies emphasize the need to take TCS into account as a possible risk factor for metabolic diseases. The probable connection between TCS exposure and IR has been explained by several different mechanisms given below.

Disruption of Gut Flora

The makeup and operation of the gut microbiome may be disrupted as a result of TCS exposure. Diabetes development has been linked to changes in the gut flora [73]. Studies suggest that TCS can cause microbial imbalances, which may help explain IR [74].

Thyroid Hormone Disruption

Thyroid hormones, which control metabolism, are known to be disrupted by TCS. TCS exposure has been demonstrated to influence thyroid hormone levels in animal tests, and hypothyroidism has been linked to T2DM [75,76].

Potential Obesity-Related Effects

TCS exposure may be associated with overweight and obesity, both of which are risk factors for T2DM. Although research on the connection between TCS exposure and body mass index (BMI) has produced mixed results, the possibility that TCS may have an impact on weight and body composition calls for additional study [72,77].

Gender Differences and Estrogenic Effects

These investigations have revealed an intriguing gender-specific link between TCS exposure and the risk of T2DM, especially in women [78]. Given that women often use more TCS-containing consumer items, variations in exposure levels may help to explain this result. TCS also demonstrates estrogen-like characteristics, which could also have implications on gender-specific health [79]. 

Environmental EDCs

Recent studies have revealed a possible connection between IR and associated metabolic abnormalities and exposure to certain environmental EDCs. This section examines the function of several EDCs, such as pesticides, heavy metals, and PAHs, in the context of IR and its corresponding health implications. PAHs are a group of chemicals composed of carbon and hydrogen atoms. They are ubiquitous environmental toxins that may be found in soil, water, and the air. Incomplete combustion processes including coal, oil, gas, car emissions, and tobacco smoke result in the production of PAHs. These substances have a high concentration in places close to industrial facilities, municipal waste incinerators, and areas with a lot of traffic [80]. Notably, PAHs are also present in common home products including rubber, coatings, and cosmetics [80].

Studies examining the relationship between urinary PAH metabolites and the development of diabetes have yielded intriguing findings. The highest levels of urine naphthalene, fluorine, phenanthrene, and total PAH metabolites were associated with considerably greater risks of T2DM, according to a comprehensive review and meta-analysis [81]. Additionally, there are links between exposure to PAHs resulting in high blood pressure and obesity [82]. Moreover, investigations using data from the NHANES have provided evidence of links between exposure to PAHs and obesity, T2DM, dyslipidemia, and hypertension [83,84].

Pesticides, such as carbamates, pyrethroids, organochlorines, and compounds of organophosphorus, are frequently employed substances having variable degrees of endocrine-disrupting potential [85]. Ingestion, inhalation, and skin absorption are the three main ways that humans are exposed to pesticides, with pesticide residues in food being a substantial source of exposure. Infants, children, expectant mothers, agricultural laborers, and pesticide applicators are among the vulnerable groups [85]. A link may exist between non-persistent pesticide exposure and diabetes or other glucose-related disorders, such as IR and β-cell dysfunction [86,87]. MetS has been examined as an individual health outcome in several studies. Regarding their relationships with excessive adiposity, dyslipidemia, and IR, studies on organochlorine pesticides have shown conflicting results [88,89]. However, it is accepted that exposure to organochlorine pesticides during pregnancy might increase the risk of metabolic abnormalities.

The Earth's crust naturally contains heavy metals including arsenic, mercury, and cadmium, which can be found in a variety of environmental sources. Tobacco smoke, tainted food, drinking water, occupational activity, and exposure to certain metals can all cause exposure. Arsenic comes in a variety of forms, and each form and species has a varied level of toxicity. The poisonous forms of arsenic are inorganic arsenic and its metabolites, such as monomethylarsonic acid (MMA) and dimethylarsinic acid (DMA). The major sources of arsenic exposure include contaminated food, water, and industrial activities. Pregnant women and occupational workers are vulnerable groups. Depending on the amount, type, and route of exposure, there is no clear link between arsenic exposure and obesity. There is some evidence linking arsenic exposure to T2DM, glucose imbalances, and these conditions. Exposure to arsenic has been positively linked to hypertension, particularly in females. Limited research suggests that in-utero exposure to arsenic may influence the development of diabetes and that there may be correlations between maternal arsenic exposure and gestational diabetes [90].

Mercury is a naturally occurring element found in air, water, and soil. Mercury exposure may come from a variety of things, such as smoking, eating seafood, and using industrial procedures. Populations at risk include fetuses and those who have occupational exposure. Studies linking obesity, glucose issues, dyslipidemia, and atherosclerosis to mercury exposure and metabolic abnormalities provide conflicting evidence [9]. Mercury may have harmful effects on blood pressure that are more likely to affect women. Cadmium is found in both natural and anthropogenic sources, with contamination often linked to industrial activities. Food can expose people to cadmium, especially in areas where the soil is cadmium-rich. Pregnant women and employees exposed to cadmium are vulnerable groups. Studies linking cadmium exposure to obesity have produced conflicting findings [91]. Evidence, however, points to possible links between cadmium exposure and concerns with glucose regulation, hypertension, and dyslipidemia [92]. There is some evidence that prenatal cadmium exposure may raise the risk of gestational diabetes [93].

## Conclusions

The intricate relationship between EDCs and IR is a complex and compelling field of study with significant implications for public health. EDCs, including phthalates, PFAS, BPA, and TCS, have been linked to IR, T2DM, and metabolic disorders. Phthalates, commonly found in consumer products, exhibit clear associations with IR, supported by epidemiological and animal studies. PFAS, while showing some conflicting findings, emphasizes the importance of specific PFAS molecules and population characteristics in influencing T2DM risk markers. BPA, present in plastics and resins, has emerged as a disruptor of glucose regulation and IR, with human and animal studies revealing its effects. TCS, found in personal care products, displays gender-specific associations with T2DM risk, potentially impacting gut microbiota and thyroid hormones. Furthermore, environmental EDCs like PAHs, pesticides, and heavy metals have also demonstrated links to T2DM, IR, hypertension, and obesity. Overall, EDCs pose a complex and evolving public health challenge, contributing to global health issues related to IR, T2DM, and obesity. Understanding the intricate molecular mechanisms behind EDC-IR interactions is crucial for developing effective prevention and intervention strategies. Further research is necessary to fully comprehend these complexities and inform policies aimed at reducing EDC exposure and preserving metabolic health.
